# Efficacy and Safety of Direct Oral Anticoagulants Versus Warfarin in Saudi Patients With Atrial Fibrillation

**DOI:** 10.7759/cureus.58886

**Published:** 2024-04-24

**Authors:** Mosaad Almegren

**Affiliations:** 1 Department of Medicine, College of Medicine, Imam Mohammad Ibn Saud Islamic University, Riyadh, SAU

**Keywords:** warfarin, rivaroxaban, apixaban, dabigatran, atrial fibrillation

## Abstract

Introduction: Atrial fibrillation (AF) significantly heightens stroke risk, which can be mitigated through anticoagulation therapy. Although warfarin was traditionally employed for this purpose, the use of direct-acting oral anticoagulants (DOACs) is on the rise.

Methods: This retrospective study, which spanned from June 2016 to January 2018, focused on adult patients diagnosed with AF. Their treatments, either via warfarin or DOACs (apixaban, rivaroxaban, and dabigatran), were evaluated. Data analysis was done using Statistical Product and Service Solutions (SPSS, version 21; IBM SPSS Statistics for Windows, Armonk, NY). This study aims to evaluate the safety and effectiveness of DOACs versus warfarin in preventing thromboembolic complications among Saudi patients with AF.

Results: A total of 396 patients with AF, averaging 66 ± 14 years of age, were part of the study. Among them, there were slightly more female patients (205 or 51.8%). The majority of patients (223 or 56.3%) were treated with a DOAC, while the rest (173 or 43.7%) received warfarin. Furthermore, 93 patients (23.5%) were taking anti-platelet drugs. Statistically, the rate of ischemic stroke was significantly higher among patients treated with DOACs than with warfarin (p=0.005), but bleeding rates were similar in both groups. Specifically, the DOACs apixaban and rivaroxaban showed a significant association with the occurrence of stroke when compared to warfarin (p=0.012 and p=007, respectively).

Conclusion: Overall, both DOACs and warfarin presented similar results regarding hemorrhagic complications when treating AF patients. However, the DOACs apixaban and rivaroxaban displayed higher risks of ischemic stroke compared to warfarin.

## Introduction

Atrial fibrillation (AF) is a common arrhythmia, affecting approximately 0.51% of people worldwide [1]. It significantly raises the risk of thromboembolic complications, notably stroke [2]. The primary treatment for AF patients to avoid these incidents is anticoagulation therapy [3].

For years, warfarin, a vitamin K antagonist, has been used to prevent stroke in AF patients [4]. A 2013 study by Björck et al. revealed a 50.4% reduction in the ischemic stroke risk in AF patients treated with warfarin [5]. The drug’s oral administration and easy availability make it advantageous. However, it also demands regular lab tests for monitoring and interacts with various other drugs [6].

In recent years, direct-acting oral anticoagulants (DOACs) emerged as an alternative to warfarin for patients with AF. Currently, the FDA has approved the DOACs rivaroxaban, apixaban, dabigatran, and edoxaban to prevent thromboembolic episodes in AF patients. This approval is backed by significant findings from randomized controlled trials [7-10]. These DOACs offer several benefits, such as oral administration, fewer hemorrhagic complications, no requirement for constant lab monitoring, and improved patient adherence [11]. However, their availability is not as widespread as warfarin, and as newer arrivals, limited data are comparing their safety and effectiveness to warfarin [12].

This study aims to evaluate the safety and effectiveness of DOACs in comparison to warfarin for anticoagulation treatment in Saudi patients with AF. We aim to disclose the clinical outcomes and complications that AF patients encounter while on anticoagulation.

## Materials and methods

This retrospective study was performed at a secondary care hospital in Riyadh, Saudi Arabia. It focused on adult patients aged 18 years and older diagnosed with AF between June 2016 and January 2018. A total of 396 patients were included in the study. AF diagnosis was determined based on echocardiogram (ECG) findings, as recorded in the electronic medical records system.

Only AF patients who were treated with DOACs such as apixaban, rivaroxaban, dabigatran, or warfarin were relevant to this study. Edoxaban was not available in our hospital at the time of the study. Patients treated with anticoagulation for reasons other than AF and pediatric patients were excluded.

Using a standardized data collection form, we gathered vital information from patient medical records. This information included demographic data, type and dosage of anticoagulants, use of antiplatelets, co-morbidities, CHADS2 score, HAS-BLED score, and outcomes, including stroke, bleeding, and death. We assessed creatinine clearance (CrCl) using the Cockcroft-Gault equation. This equation considers the patient’s gender, age, serum creatinine levels, and body weight [13].

During the initial evaluation of AF, the CHADS2 score was used to assess all patients’ risk for thromboembolic complications. This score takes into account the patient’s age, hypertension presence, congestive heart failure, diabetes mellitus, and stroke history [14]. Anticoagulation therapy is initiated based on the American Heart Association/American College of Cardiology guidelines.

The baseline HAS-BLED score was also calculated to evaluate the risk of hemorrhagic complications during anticoagulation therapy. This score takes into account factors such as the patient’s age, medication history, alcohol intake, and co-existing conditions such as hypertension, bleeding issues, stroke, and abnormal kidney and liver function [15]. A HAS-BLED score of 3 or higher indicates a significant bleeding risk.

Major bleeding was identified according to the International Society on Thrombosis and Haemostasis (ISTH) criteria [16]. This definition encompasses any bleeding episode causing death, bleeding at a crucial anatomical site such as intracranial, pericardial bleeding, or bleeding that results in a 2 g/dL drop in hemoglobin levels or the need for a transfusion of two units of packed red blood cells.

Data were evaluated utilizing Statistical Product and Service Solutions (SPSS, version 21; IBM SPSS Statistics for Windows, Armonk, NY). For quantitative variables (such as age, CrCl, CHADS2, and HAS-BLED scores), the mean and standard deviation were calculated. Frequencies and percentages were determined for qualitative variables such as gender, co-morbidities, anticoagulation therapy, and complication occurrence. The Pearson chi-square test and Mann-Whitney U tests compared the outcomes of patients treated with DOACs and those treated with warfarin. A P value of less than 0.05 was deemed statistically significant for all data analyses.

The hospital Ethics Review Committee approved the commencement of this study. Given its retrospective design, it required no funding.

## Results

This study included 396 patients diagnosed with AF, of which 223 (56.3%) received DOACs and 173 (43.7%) were treated with warfarin. Of the patients treated with DOACs, 138 (61.8%) received apixaban, 40 (17.9%) were assigned dabigatran, and 45 (20.2%) had rivaroxaban therapy. The participants' mean age was 66±14 years. Out of them, 191 (48.2%) were males, and 205 (51.8%) were females. They were followed up for a median duration of 12 months, ranging from 0 to 39 months (Table 1).

**Table 1:** Baseline characteristics of patients who were treated with DOACs compared to those who received warfarin. P value is considered significant (P<0.05). DOACs: direct-acting oral anticoagulants

Baseline characteristics	Total, N = 396 (%)	DOACs, N = 223 (%)	Warfarin, N = 173 (%)	P value
Age (years), mean ± SD	66 ± 14	69 ± 14	63 ± 15	<0.001
Males, N (%)	191 (48.2)	97 (50.8)	94 (49.2)	0.032
Weight (kg) mean ± SD	80 ± 19	81 ± 19	79 ± 19	0.238
Comorbidities: DM	217 (54.8)	120 (55.3)	97 (44.7)	<0.001
HTN	258 (65.2)	152 (58.9)	106 (41.1)	<0.001
Heart failure	135 (34.2)	71 (52.6)	64 (47.4)	0.336
Ischemic stroke	74 (18.7)	46 (62.2)	28 (37.8)	0.299
Hemorrhagic stroke	20 (5.1)	19 (95)	1 (5)	0.001
ESRD	38 (9.6)	12 (31.6)	26 (68.4)	0.001
CHADS score, mean ± SD	2 ± 1.2	2.3 ± 1.3	1.7 ± 1.0	0.009
HAS-BLED score, mean ± SD	1.7 ± 1.2	2.0 ± 1.3	1.3 ± 0.8	<0.001
Creatinine clearance, mean ± SD	78 ± 40	84 ± 36	69 ± 44	0.012
ASA	76 (19.2)	45 (59.2)	31 (40.8)	0.571
Plavix	11 (2.8)	9 (81.8)	2 (18.2)	0.123
ASA+Plavix	6 (1.5)	5 (83.3)	1 (16.7)	0.238
Platelet count (x 10^9^/uL)	258 ± 106	257 ± 114	258 ± 96	0.708
Hemoglobin (g/dL)	12.7 ± 2.5	13.2 ± 2.6	12.0 ± 2.3	0.007

Hypertension was the most common comorbidity present in 65.2% (258) of the patients. Other notable conditions were diabetes mellitus in 54.8% (217), heart failure in 34.2% (135), end-stage renal disease in 9.6% (38), ischemic stroke in 18.7% (74), and hemorrhagic stroke in 5.1% (20) patients. Additionally, 23.5% (93) of the patients were also taking anti-platelet medications. Both the baseline CHADS2 and HAS-BLED scores were significantly higher in patients using DOACs compared to those treated with warfarin (p=0.009 and p<0.001, respectively). Hemorrhagic stroke history also significantly increased in the DOACs group (p=0.001). The patient’s renal function, determined by creatinine clearance, was considerably lower in patients prescribed warfarin compared to those on DOACs (p=0.012).

**Figure 1:**
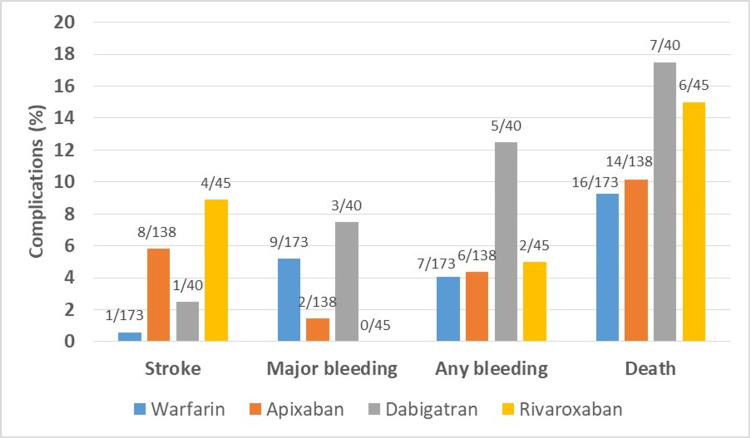
Complications with anticoagulation therapy, reported as a percentage of outcomes from the total number of patients who received each drug. The data are represented as numbers and percentages.

In total, 13 patients (5.8%) on DOACs were diagnosed with new ischemic stroke compared to only one patient (0.6%) on warfarin (p=0.05). Major bleeding, any bleeding, and mortality showed similar rates across both groups. Table 2 examines the outcomes of patients administered DOACs versus those given warfarin. Out of the 396 total patients, 43 (10.8%) died during the study. Figure 1 underscores the complications in our study group, categorized according to anticoagulation type.

**Table 2:** Outcomes of patients received DOACs vs. warfarin. The data are represented as numbers and percentages. P value is considered significant (p<0.05).

	DOAC (n=223), N (%)	Warfarin (n=173), N (%)	P value
Stroke	13 (92.9)	1 (7.1)	0.005
Major bleeding	5 (45.5)	6 (54.5)	0.544
Any bleeding	13 (59.1)	9 (40.9)	0.787
Death	27 (62.8)	16 (37.2)	0.333

The DOACs apixaban and rivaroxaban exhibited a significant association with ischemic stroke compared to warfarin (p=0.012 and p=0.007, respectively). However, the rates of major and any bleeding were comparable between these groups. Dabigatran showed no significant difference from warfarin in terms of patient outcomes.

Tables 3-4 summarize the outcomes in patients who were treated with apixaban and rivaroxaban compared to those who received warfarin.

**Table 3:** Comparison of outcomes in patients who received apixaban vs. warfarin. The data are represented as numbers and percentages. P value is considered significant (p<0.05).

	Apixaban (n=138), N (%)	Warfarin (n=173), N (%)	P value
Stroke	8 (88.9)	1 (11.1)	0.012
Major bleeding	2 (25)	6 (75)	0.308
Any bleeding	6 (40)	9 (60)	0.727
Death	14 (46.7)	16 (53.3)	0.726

**Table 4:** Comparison of outcomes in patients who received rivaroxaban vs. warfarin. The data are represented as numbers and percentages. P value is considered significant (p<0.05).

	Rivaroxaban (n=45) N (%)	Warfarin (n=173) N (%)	P value
Stroke	4 (80)	1 (20)	0.007
Major bleeding	0 (0)	6 (100)	0.349
Any bleeding	2 (18.2)	9 (81.8)	1.000
Death	6 (27.3)	16 (72.7)	0.418

The assessment of patients treated with DOACs was conducted to determine if they received an appropriate dosage according to FDA guidelines. Out of 223 patients receiving DOACs, 71.7% (160) received an appropriate dose based on FDA recommendations, while 28.3% (63) received an inappropriate one considering age, weight, and renal function. A comparison between the 160 patients on an appropriate DOAC dose and the 173 patients on warfarin revealed a significant relation with stroke for DOACs (p=0.031). Despite higher risks of major bleeding and any bleeding linked to warfarin use, our data did not reach statistical significance (p=0.28, p=0.34). When the 63 patients who received an inappropriate DOAC dose were compared to the 173 warfarin users, it was disclosed that inappropriate DOAC dosage again posed a significant stroke risk (p=0.002).

## Discussion

This study evaluated the safety and effectiveness of DOACs in comparison to warfarin in treating Saudi patients with AF. Overall, DOACs were linked with an increased risk of ischemic stroke compared to warfarin. However, both had similar rates of bleeding complications and mortality. According to numerous prior reports, there is no significant difference between DOACs and warfarin in terms of overall mortality [17,18]. However, a recent study showed that, in the long term, AF patients using DOACs may have a better survival rate than those on warfarin [19]. Separately, a study involving over 100,000 AF patients from the UK offered mixed results. It indicated that the DOAC apixaban had the fewest bleeding complications. Contrarily, the mortality rate was higher for patients on rivaroxaban and low-dose apixaban compared to those on warfarin [20].

The DOACs apixaban and rivaroxaban have shown an increased risk of stroke compared to patients treated with warfarin. In our study, this elevated stroke risk associated with DOACs may be attributed to underlying patient characteristics. Specifically, patients receiving DOACs were older and had higher baseline CHADS and HAS-BLED scores than those prescribed warfarin. Additionally, the comparatively short half-life of DOACs could contribute to the increased stroke risk.

A secondary analysis of the ROCKET AF clinical trial found similar rates of stroke and thromboembolic complications among AF patients treated with rivaroxaban and warfarin [21]. Conversely, a study by Gupta et al. reported a lower stroke risk among AF patients treated with the DOAC apixaban compared to those treated with warfarin [22]. Another large-scale study, encompassing over 100,000 AF patients, indicated that DOACs were associated with a higher risk of stroke compared to warfarin. This trend persisted when all four DOACs - rivaroxaban, apixaban, dabigatran, and edoxaban - were compared to warfarin [23].

In the same study, it was suggested that warfarin’s pharmacological properties, particularly its longer duration of action, might lead to more effective anticoagulation and a lower risk of ischemic stroke compared to DOACs [23]. Further investigations involving patients of diverse age groups, weights, and co-morbidities are necessary to reconcile these discrepancies in the literature.

No significant difference in major or any bleeding episodes was observed among our patients receiving DOACs and warfarin. It contradicts a meta-analysis involving over 2.3 million AF patients, where DOACs were linked to a lower risk of hemorrhagic complications compared to warfarin treatment [24]. A further subgroup analysis on an AF patient cohort revealed that, among DOACs, apixaban and dabigatran were specifically associated with a lower likelihood of bleeding than warfarin. However, this association was not found with rivaroxaban use [25].

Patients treated with warfarin showed a markedly lower baseline creatinine clearance compared to those who received DOACs. Warfarin has been the drug of choice for anticoagulants in patients with end-stage renal disease (ESRD) [26]. However, in a randomized controlled trial by Stanifer et al., it was found that, in patients with AF and CrCl between 25 and 30 mL/min, the DOAC apixaban resulted in fewer bleeding episodes than warfarin [27]. Additionally, a recent systematic review found similar safety and effectiveness between warfarin and DOACs in AF patients with kidney disease and a CrCl range of 15-60 mL/min [28].

This study has limitations, including its retrospective, single-center nature and relatively small sample size, which may not adequately power the comparisons between DOACs and warfarin. Further research, through larger retrospective studies and prospective studies, is needed to effectively evaluate the efficacy and safety of different anticoagulation medications in the Saudi population with AF.

## Conclusions

In our study of Saudi patients with AF, both warfarin and DOACs (including apixaban, dabigatran, and rivaroxaban) showed comparable rates of bleeding events and all-cause mortality. However, DOACs presented a higher risk of ischemic stroke than warfarin.
